# Clinical staging and the differential risks for clinical and functional outcomes in young people presenting for youth mental health care

**DOI:** 10.1186/s12916-022-02666-w

**Published:** 2022-12-14

**Authors:** William Capon, Ian B. Hickie, Mathew Varidel, Ante Prodan, Jacob J. Crouse, Joanne S. Carpenter, Shane P. Cross, Alissa Nichles, Natalia Zmicerevska, Adam J. Guastella, Elizabeth M. Scott, Jan Scott, Jai Shah, Frank Iorfino

**Affiliations:** 1 Brain and Mind Centre, The University of Sydney, Sydney, 2050 Australia; 2 Translational Health Research Institute, Western Sydney University, Sydney, 2751 Australia; 3 School of Computer, Data and Mathematical Sciences, Western Sydney University, Sydney, 2751 Australia; 4 School of Psychological Sciences, Faculty of Medicine, Health and Human Sciences, Macquarie University, Sydney, 2109 Australia; 5 Academic Psychiatry, Institute of Neuroscience, Newcastle University, Newcastle upon Tyne, NE1 7RU UK; 6 Department of Psychiatry, McGill University, Montreal, H3A 0G4 Canada

**Keywords:** Young people, Mental health, Multidimensional outcomes, Clinical stage, Risk

## Abstract

**Background:**

Clinical staging proposes that youth-onset mental disorders develop progressively, and that active treatment of earlier stages should prevent progression to more severe disorders. This retrospective cohort study examined the longitudinal relationships between clinical stages and multiple clinical and functional outcomes within the first 12 months of care.

**Methods:**

Demographic and clinical information of 2901 young people who accessed mental health care at age 12–25 years was collected at predetermined timepoints (baseline, 3 months, 6 months, 12 months). Initial clinical stage was used to define three fixed groups for analyses (stage 1a: ‘non-specific anxious or depressive symptoms’, 1b: ‘attenuated mood or psychotic syndromes’, 2+: ‘full-threshold mood or psychotic syndromes’). Logistic regression models, which controlled for age and follow-up time, were used to compare clinical and functional outcomes (role and social function, suicidal ideation, alcohol and substance misuse, physical health comorbidity, circadian disturbances) between staging groups within the initial 12 months of care.

**Results:**

Of the entire cohort, 2093 young people aged 12–25 years were followed up at least once over the first 12 months of care, with 60.4% female and a baseline mean age of 18.16 years. Longitudinally, young people at stage 2+ were more likely to develop circadian disturbances (odds ratio [OR]=2.58; CI 1.60–4.17), compared with individuals at stage 1b. Additionally, stage 1b individuals were more likely to become disengaged from education/employment (OR=2.11, CI 1.36–3.28), develop suicidal ideations (OR=1.92; CI 1.30–2.84) and circadian disturbances (OR=1.94, CI 1.31–2.86), compared to stage 1a. By contrast, we found no relationship between clinical stage and the emergence of alcohol or substance misuse and physical comorbidity.

**Conclusions:**

The differential rates of emergence of poor clinical and functional outcomes between early versus late clinical stages support the clinical staging model's assumptions about illness trajectories for mood and psychotic syndromes. The greater risk of progression to poor outcomes in those who present with more severe syndromes may be used to guide specific intervention packages.

**Supplementary Information:**

The online version contains supplementary material available at 10.1186/s12916-022-02666-w.

## Background

Mental disorders are a leading cause of disability worldwide and the burden on young people continues to increase [1, 2]. The excess morbidity and mortality appear to be driven by two overlapping phenomena. First, 75% of adult-pattern mental disorders emerge by the age of 25 [3], with psychiatric comorbidity the rule rather than the exception even in this young age group [4]. Second, presentations of full- or sub-threshold syndromes are typically accompanied by other impairments across multiple clinical and functional domains, including vocational and educational instability, suicidality, alcohol or substance misuse, physical health comorbidity, and sleep-wake cycle disturbance [5]. Overall, the complexity and heterogeneity of the presentation and evolution of mental disorders during adolescence and early adulthood have not only complicated treatment planning, but also exposed significant concerns regarding the reliability, validity, and applicability of current diagnostic approaches which employ traditional, symptom-based criteria [5, 6].

To address these problems, a multidimensional outcomes framework has been developed to highlight the diverse needs of young people who present to mental health care and to guide interventions which target traditionally defined disorders and their broader impacts [5]. The five domains of the framework are social and occupational function, self-harm and suicidality, alcohol or substance misuse, physical health, and illness type, stage, and trajectory. The known effect of poor mental health on these domains demonstrates the benefit of multidimensional assessment in youth mental health care [7–12]. For example, vocational disengagement (‘not in education, employment, and training’) (NEET) is almost twice as common in youths seeking mental health care (19%) compared with the general population (11%) [7]. Similarly, approximately one in three young people seeking care have already experienced suicidal thoughts compared with an estimated 8% of the general adolescent population [8, 12], and approximately one-third misuse alcohol or other substances (e.g. nicotine, cannabis) [10].

One key component of the multidimensional framework is assigning a clinical stage to the clinical presentation, alongside formal diagnosis [13]. The clinical staging model for mood and psychotic disorders was designed and integrated into youth mental health care to recognise illness progression and guide clinical decision-making, which includes choosing the types and intensities of interventions best suited to individual needs [5, 6]. This approach is transdiagnostic and acknowledges that individuals can be located on a broad continuum, typically spanning from early to later-stage illness [6]. The early stages, 1a (nonspecific symptoms) and 1b (attenuated syndromes and so-called at-risk or clinical high-risk states), are more easily differentiated from later stages (stage 2+), since stage 2 demarcates the onset of discrete, full-threshold, and significant disorders (and stages 3 and 4 represent established long-term disorders) [14]. Successive stages are more likely to experience illness progression (i.e., stage transitions) to the next stage and less likely to remit [15] — thereby requiring more intensive treatment options with a higher risk-to-benefit ratio. Furthermore, recognised clinical risk factors (including psychotic- and manic-like experiences and circadian disturbances) are associated with future stage progression [15].

The theoretical utility of a staging model, particularly if adopted in youth primary care services, has been recently outlined as a promising step towards better identification and treatment of disorders [16–18]. This includes the potential to predict the onset of new clinical and functional outcomes, including multidimensional factors, to justify the early allocation of more intense and specific interventions for those at greater risk. One potential marker of clinical risk is clinical stage, yet there is a need to test the assumption that staging distinguishes the risk of poor multidimensional outcomes emerging overtime.

The present study therefore tests the assumption that illness progression is associated with the onset of poor clinical and functional outcomes in a cohort of young people who presented to mental health care within a 12-month period. We aim to identify the different rates of emergence of poor social and occupational functioning, suicidal ideation, alcohol or substance misuse, physical health comorbidities, and circadian disturbances (representing each domain of the multidimensional outcomes framework) between clinical staging groups.

## Methods

### Ethical considerations

This study was approved by the University of Sydney Human Research Ethics Committee, Australia (number 2012/1626) and written informed consent for the use of routinely collected data was given by participants, their guardians, or both.

### Participants

This was a retrospective cohort study and STROBE guidelines were abided by. Participants were drawn from a cohort of 2901 individuals aged 12 to 30 years who presented and registered to youth mental health clinics in Sydney, Australia, affiliated with the Brain and Mind Centre (University of Sydney), between June 2008 and July 2018 [15, 19]. The clinics included primary care services (including *headspace* [20], a clinic for young people seeking mental, physical, and/or sexual health care) and specialist psychiatric services, wherein individuals may be self-referred, or referred by family, friends, or members of the community (including medical practitioners, universities) [21]. All participants received standard care, including clinician-based case management and psychological, medical, and/or social interventions that were appropriate for the individual case.

### Eligibility criteria

Individuals included for analyses were (i) between 12 to 25 years of age at the time of initial assessment and (ii) followed up at least once between 1 to 12 months after initial assessment.

### Data collection

Using a clinical research proforma, demographic, clinical, and functional information from clinical files, research files, and code inputs were extracted by trained research staff. All clinical notes were generated by the study participants’ treating clinician(s) as part of their standard care. The proforma recorded data at predetermined time points relative to the first available clinical assessment at the service (hereinafter referred to as the ‘baseline’). These follow-up timepoints were set for research purposes at 3 months, 6 months, 12 months, 2 years, 3 years, 4 years, and 5 years. For individuals that did not attend the service at a particular time point, that entry was left missing. Data from preceding timepoints were used to inform and complete subsequent entries.

### Assessments/clinical proforma

Detailed description and an inter-rater reliability analysis of the proforma have been previously published [19] and additional information about the data collection and variables used in this study are shown in Additional file 1 [13–15, 22–33]. The clinical stage was assessed according to the model used by Hickie et al. [14], and assigned at each timepoint alongside formal diagnoses. Specifically, stage 4 (the most severe illness stage) constitutes chronic debilitating illnesses, stage 3 is embodied by persistent or recurrent illnesses, and stage 2 is assigned to individuals with discrete disorders. Therefore, young people with full threshold disorders that are major, discrete, and persistent are assigned stage 2+, including clear episodes of psychosis, mania, or severe depression. Young people with attenuated syndromes were assigned to the stage 1b group. These are considered less severe or intermittent syndromes compared to stage 2+. Young people assigned to stage 1a usually have fewer symptoms and milder impairment compared to stage 1b, and these presentations typically do not meet diagnostic criteria.

To analyse the relationship between clinical stage upon entry to care and future outcomes, individuals were fixed in their staging group (1a, 1b, 2+) according to their stage upon initial presentation (regardless of potential stage transitions). We selected six variables for longitudinal analysis (NEET status, Social and Occupational Functioning Assessment Scale (SOFAS; [22]) score, circadian disturbances, suicidal ideation, alcohol or substance misuse, physical health comorbidity) to encapsulate the five domains of the multidimensional framework [5]. NEET status was assigned if the individual was not involved in any education, employment, training, or volunteer work at presentation for care [19]. A clinician-administered SOFAS 22) score between 0 and 100 was also recorded, with a score of below 70 indicating poor functioning. Based on clinical notes, the presence of circadian disturbances was recorded using a high threshold (significant disruption in circadian rhythm sleep-wake cycles, including chronic fatigue or a severe sleep-wake disorder that impacted daily functioning). Suicidal ideation (thoughts of self-harm or intentional consideration of performing suicide), alcohol or substance misuse (the presence of a substance-related disorder according to DSM-5 criteria), and major physical illness (the diagnosis of any physical health comorbidity, e.g. respiratory illness, endocrine illness) were also recorded by interpreting clinical notes. For the baseline timepoint, the research staff coded for variables based on the participant’s lifetime (e.g. suicidal ideation was recorded as present if the individual had any history of suicidal ideation). Subsequent timepoints (i.e. follow-ups) were coded based on what had occurred between timepoints (e.g. for timepoint X, suicidal ideation was recorded as present if the individual had suicidal thoughts between timepoint X and timepoint X-1).

### Statistical analyses

All analyses were performed using R statistical software (R Foundation, version 3.5.1). A priori, we determined that all individuals with a mental disorder that met full-threshold diagnostic criteria (in the clinical staging model these presentations may be assigned as stages 2, 3, and 4) would be included in a single group which we named 'stage 2+', to avoid multiple comparisons between small subgroups. Pairwise comparisons of baseline demographic and clinical characteristics for each clinical staging group were computed using a one-way analysis of variance (ANOVA) and post hoc Tukey-Kramer tests for pairwise comparisons of continuous variables, and Pearson’s chi-square (*χ*^2^) tests for categorial variables. One-way ANOVA and post hoc Tukey-Kramer tests with pairwise comparisons were also used to compare the median follow-up time and number of assessments for each staging group to identify any sources of bias that may occur from loss to follow-up. An initial alpha level of 0.05 was chosen for all analyses and an adjusted alpha level of 0.001 was used according to a Bonferroni correction.

Logistic regression models were used to examine the association between the clinical stage and the emergence of multidimensional outcomes up to 12 months after the initial assessment. Emergence was defined as the presence of any outcome that was initially absent at baseline. The models computed the relative odds for each outcome emerging at any follow-up assessment within 12 months of baseline (e.g. NEET, suicidal ideation) between the clinical staging groups. For each logistic regression model, we excluded individuals who initially had the presence of the variable of interest and controlled for age and follow-up time. The odds ratios (ORs) and 95% confidence intervals (CIs) were reported for each pairwise comparison of the three clinical staging groups, as well as the sample sizes for each logistic regression model.

## Results

### Sample characteristics

**Fig. 1:**
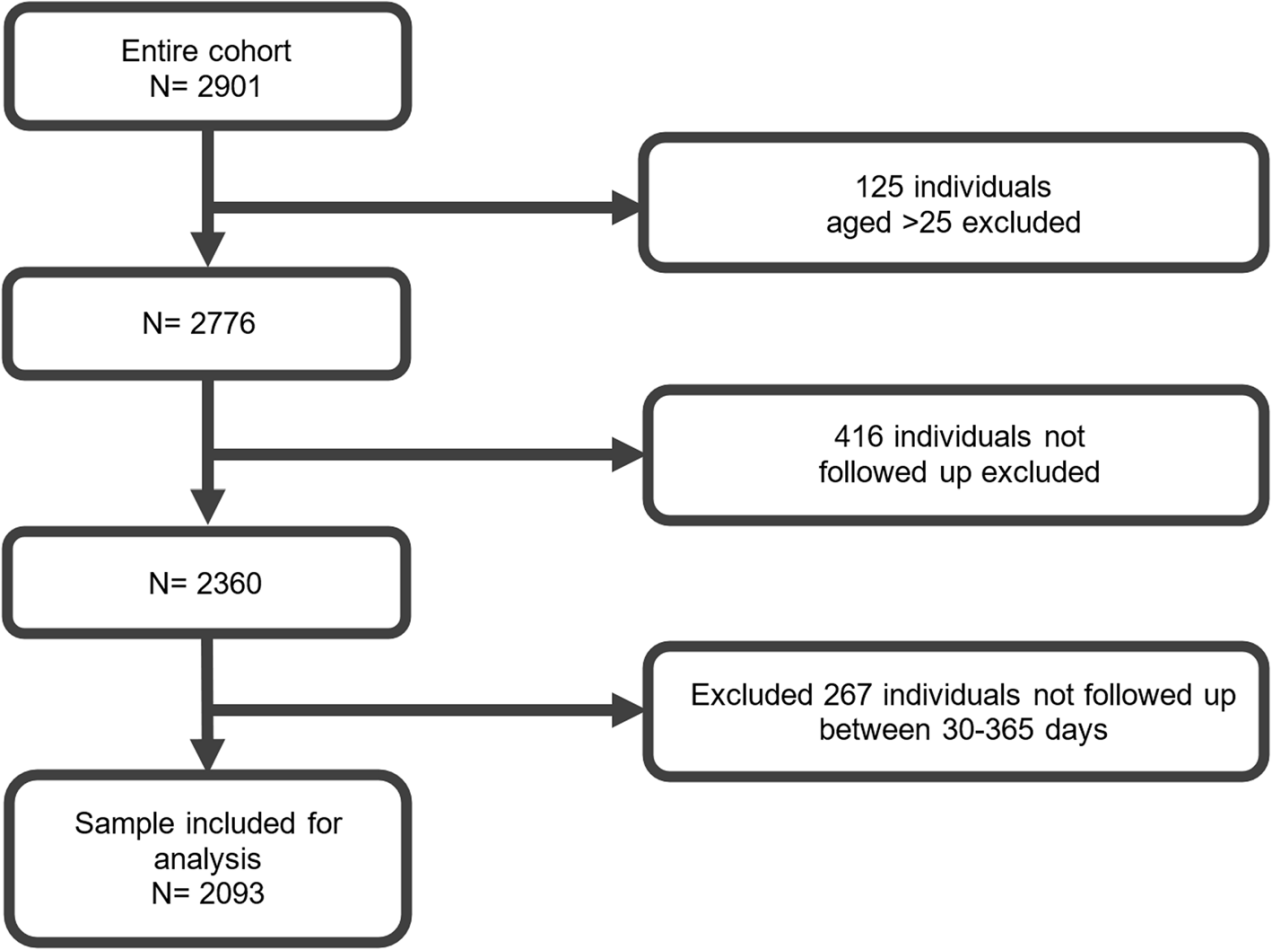
Study flow diagram. Eligible individuals at each step of the inclusion criteria

The cohort comprised 2093 individuals (Fig. 1); 60.4% were female with a baseline mean age of 18.16 years (standard deviation, SD 3.32). Baseline characteristics and pairwise comparisons for each clinical staging group are shown in Table 1. The median follow-up time was 183 days (range, 32–364) and the median number of observations per participant was 3 (range, 2–4). There were no differences in the number of observations (*F*_2,2090_=1.92; *P*=0.15) or follow-up time (*F*_2,2090_=4.26; *P*=0.014) between clinical staging groups. As shown in Fig. 1, of the entire cohort (*n*=2901), 683 individuals were not followed up between 30 and 365 days (416 lost to follow-up and 267 followed up outside of this range). There were some differences in follow-up rates between the three staging groups (*F*_2,2776_=68.259; *P*<0.001). Specifically, stage 2+ were more likely to not be followed up compared to those in stage 1b (44.22% lost to follow-up *v* 22.44%; *χ*^2^(1)=60.80; *P*<0.001) and stage 1a (44.22% *v* 21.97%; *χ*^2^(1)=52.52; *P*<0.001), yet there was no difference in loss to follow-up rates between stages 1a and 1b (21.97% *v* 22.44% *χ*^2^(1)=0.05; *P*=0.83).

**Table 1:** Baseline demographic and clinical characteristics of individuals by clinical stage (*N* = 2093)

	Number of individuals				Comparison^**a**^		
	Group						
**Characteristic**	**Stage 1a**	**Stage 1b**	**Stage 2+**	***P*** **value**^**a**^	**1b v 1a**	**2+ v 1a**	**1b v 2+**
Stage at baseline, (%)	650 (31.1)	1279 (61.1)	164 (7.8)	NA	NA	NA	NA
Mean age (y), (SD)	17.13 (3.26)	18.41 (3.22)	20.30 (2.90)	<0.001	<0.001	<0.001	<0.001
Female (%)	383 (58.9)	795 (62.2)	87 (53.0)				
*Social and occupational functioning*							
Mean SOFAS score, (SD)^b^	66.96 (8.14)	60.86 (8.34)	55.91 (10.29)	<0.001	<0.001	<0.001	<0.001
NEET (%)	49 (7.5)	227 (17.7)	58 (35.4)	<0.001	<0.001	<0.001	<0.001
*Clinical presentation (%)*							
Manic-like experiences	22 (3.4)	229 (17.9)	70 (42.7)	<0.001	<0.001	<0.001	<0.001
Psychotic-like experiences	42 (6.5)	256 (20.0)	86 (52.4)	<0.001	<0.001	<0.001	<0.001
Circadian disturbance	43 (6.6)	208 (16.3)	59 (36.0)	<0.001	<0.001	<0.001	<0.001
Neurodevelopmental - ASD	25 (3.8)	58 (4.5)	7 (4.3)				
Neurodevelopmental - ADHD	57 (8.8)	111 (8.7)	8 (4.9)				
Neurodevelopmental - other	22 (3.4)	25 (2.0)	1 (0.6)				
*Personal history of mental illness (%)*							
Any childhood disorder	70 (10.8)	202 (15.8)	24 (14.6)				
Any family history	262 (40.2)	656 (51.3)	89 (54.3)	<0.001	<0.001		
*Physical health comorbidities (%)*							
Any major physical illness	96 (14.8)	193 (15.1)	37 (22.6)				
*Alcohol and/or substance misuse (%)*							
Any alcohol and/or substance misuse	17 (2.6)	128 (10.0)	27 (16.5)	<0.001	<0.001	<0.001	
*Treatment utilisation (%)*							
Any hospitalisation	5 (0.8)	162 (12.7)	115 (70.1)	<0.001	<0.001	<0.001	<0.001
Any psychiatric medication	111 (17.1)	704 (55.0)	149 (90.0)	<0.001	<0.001	<0.001	<0.001
*Self-harm and suicidal thoughts and behaviours (%)*							
Self-harm	146 (22.5)	650 (50.8)	73 (44.5)	<0.001	<0.001	<0.001	
Suicidal ideation	194 (29.8)	733 (57.3)	101 (61.6)	<0.001	<0.001	<0.001	
Suicide attempt	17 (2.6)	231 (18.1)	54 (32.9)	<0.001	<0.001	<0.001	<0.001

*Abbreviations*: *ADHD* Attention-deficit/hyperactivity disorder, *ASD* Autism spectrum disorder, *NA* Not applicable, *NEET* Not involved in employment, education, or training, *SD* Standard deviation, *SOFAS* Social and Occupational Functional Assessment Scale, *v* versus, *y* years

^a^*P*<0.001 is the adjusted α level for statistical significance

^b^SOFAS scores for eight individuals were missing (one from stage 1a, six from stage 1b, two from stage 2+)

At baseline, those at later clinical stages were more likely to be older (*F*_2,2093_=73.91; *P*<0.001) and more likely to be functionally impaired as indexed by higher NEET rates (*χ*^2^(2)=83.47; *P*<0.001) and SOFAS scores (*F*_2,2082_=162.50; *P*<0.001). There were no differences in the proportion of neurodevelopmental disorders (autism spectrum disorder, *χ*^2^(2)=0.50; *P*=0.78; attention-deficit/hyperactivity disorder, χ^2^(2)=2.89; *P*=0.24; other, *χ*^2^(2)=6.18; *P*=0.05) or physical illnesses (*χ*^2^(2)=6.64; *P*=0.04) across the staging groups. Later clinical stages were associated with higher rates of manic-like experiences (*χ*^2^(2)=172.46; *P*<0.001), psychotic-like experiences (*χ*^2^(2)=190.91; *P*<0.001) and circadian disturbances (*χ*^2^(2)=94.96; *P*<0.001). Stage 1a was associated with lower rates of alcohol and/or substance misuse compared to stage 1b (*χ*^2^(1)=32.82; *P*<0.001) and 2+ (*χ*^2^(1)=46.44; *P*<0.001), however, there were no differences in alcohol and/or substance misuse in stages 1b and 2+ (*χ*^2^(1)=5.66; *P*=0.02). There were also different patterns of suicidality and self-harm across the staging groups. Earlier clinical stages were associated with lower rates of suicide attempts (*χ*^2^(2)=132.58; *P*<0.001), and stage 1a individuals were less likely to have engaged in self-harm behaviours compared with the 1b group (*χ*^2^(1)=141.84; *P*<0.001) and 2+ group (*χ*^2^(1)=31.27; *P*<0.001) and also less likely to have suicidal thoughts compared with the 1b group (*χ*^2^(1)=129.13; *P*<0.001) and 2+ group (*χ*^2^(1)=55.73; *P*<0.001). Yet, there were no differences between the stage 1b and 2+ groups for self-harm behaviours (*χ*^2^(1)=2.07; *P*=0.15) and the presence of suicidal thoughts (*χ*^2^(1)=0.92; *P*=0.34). Later clinical stages (1b and 2+) were also associated with increased rates of prior hospitalisation (*χ*^2^(2)=542.13; *P*<0.001) and psychiatric medication (*χ*^2^(2)=393.76; *P*<0.001) compared to stage 1a .

### Associations between clinical stages and the emergence of multidimensional outcomes

**Fig. 2:**
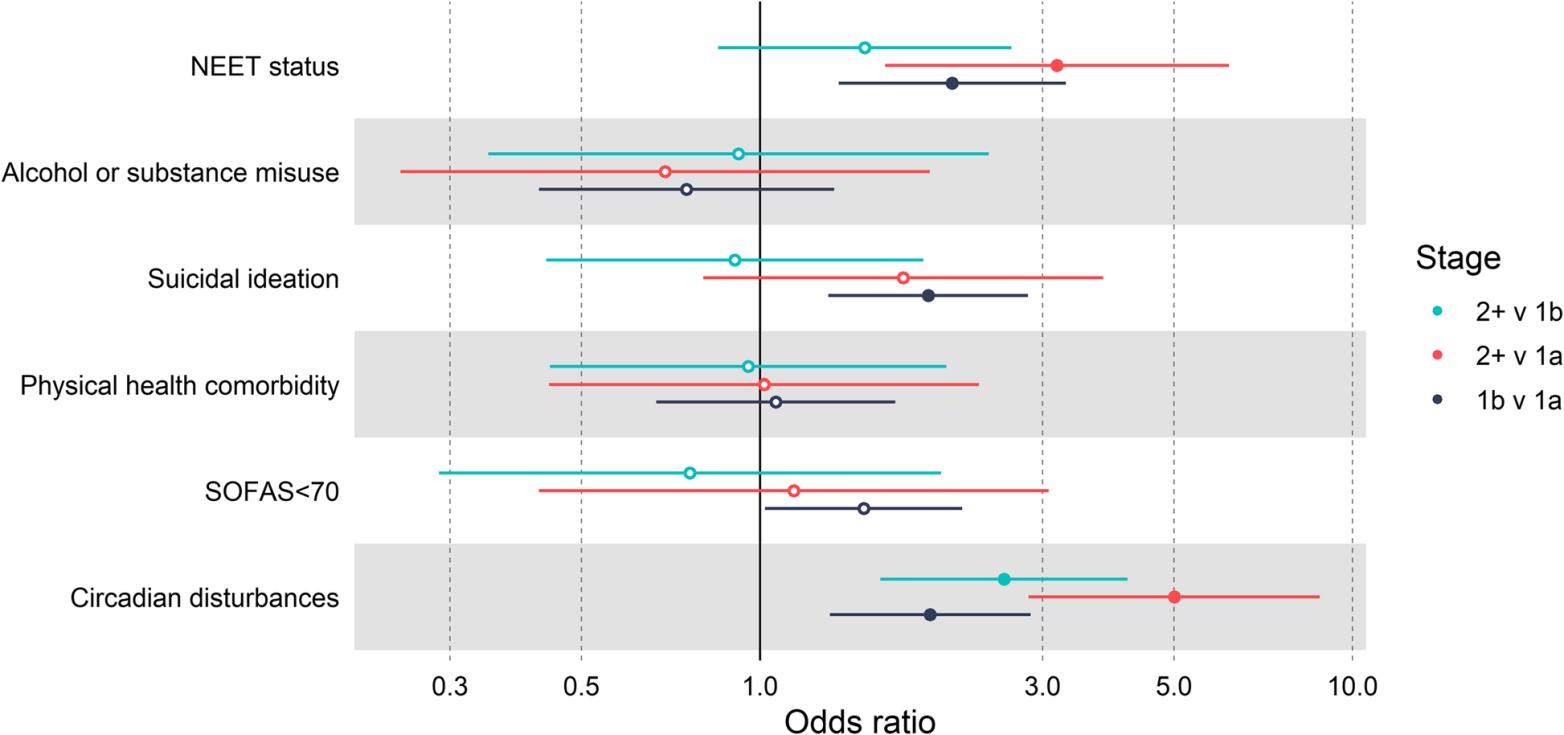
Emergence of outcomes between clinical staging groups after 12 months of mental health care. Covariates of the models included age and follow-up time. Abbreviations: NEET, not involved in employment, education, or training; SOFAS, Social and occupational functioning assessment scale; v, versus. Reference group is the earlier stage of each comparison. Odds ratios were determined using logistic regression. Filled-in circles indicate *P*<0.001, open circles indicate non-significance. Error bars represent 95% confidence intervals

The sample sizes for each logistic regression model are presented in Table 2. After controlling for age and follow-up time, stage 1b individuals were at higher risk of becoming NEET (OR=2.11, 95% CI 1.36–3.28, *P*<0.001), and developing circadian disturbances (OR=1.94, 95% CI 1.31–2.86, *P*<0.001) and suicidal ideations (OR=1.92, 95% CI 1.30–2.84, *P*<0.001) compared with those in stage 1a (Table 2, Fig. 2). Circadian disturbances were more likely to emerge in the stage 2+ group compared with stage 1b (OR=2.58, 95% CI 1.60–4.17, *P*<0.001), yet there were no other clear differences in emergence between stage 1b and 2+ groups. Young people at stage 2+ compared with stage 1a were more likely to become NEET (OR=3.17, 95% CI 1.63–6.19, *P*<0.001) and develop circadian disturbances (OR=5.00, 95% CI 2.84–8.81, *P*<0.001). The onset of poor social and occupational functioning (as indexed by SOFAS scores), alcohol or substance misuse, and physical comorbidities did not differ between the clinical staging groups after 12 months of care.

**Table 2:** Associations among clinical staging groups and emergence of multidimensional outcomes within 12 months of care

Outcome	OR (95%CI)			Number of individuals with outcome [%]		
	1b v 1a	2+ v 1a	2+ v 1b	1a	1b	2+
NEET status (*n*=1759)	2.11 (1.36–3.28)**	3.17 (1.63–6.19)**	1.50 (0.85–2.66)	27 [4.50]	103 [9.79]	17 [16.04]
Alcohol or substance misuse (*n*=1921)	0.75 (0.42–1.33)	0.69 (0.25–1.93)	0.92 (0.35–2.44)	20 [3.16]	33 [2.87]	5 [3.65]
Suicidal ideation (*n*=1065)	1.92 (1.30–2.84)**	1.75 (0.80–3.79)	0.91 (0.44–1.89)	46 [10.09]	93 [17.03]	10 [15.87]
Physical health comorbidity (*n*=1767)	1.06 (0.67–1.69)	1.02 (0.44–2.34)	0.96 (0.44–2.06)	28 [5.05]	63 [5.80]	8 [6.30]
SOFAS<70 (*n*=670)	1.50 (1.02–2.20)*	1.14 (0.42–3.07)	0.76 (0.29–2.02)	66 [20.00]	85 [26.98]	6 [24.00]
Circadian disturbances (*n*=1783)	1.94 (1.31–2.86)**	5.00 (2.84–8.81)**	2.58 (1.60–4.17)**	36 [5.93]	123 [11.48]	29 [27.61]

Covariates of the models included age and follow-up time. *Abbreviations*: *CI* Confidence intervals, *n* sample size for logistic regression model, *NEET* Not involved in employment, education, or training, *OR* Odds ratio, *SOFAS* Social and occupational functioning assessment scale, *v* versus. For each comparison using logistic regression, the reference group is the earlier stage to determine odds ratios. **P<0.001 (adjusted *α* level for statistical significance), * *P*<0.05

## Discussion

This study demonstrates that clinical stage at entry into care can effectively differentiate the risk of poor outcomes in young people within the first 12 months of mental health care. Specifically, NEET status, circadian disturbances, and suicidal ideations were more likely to emerge for individuals at later clinical stages compared to those in the earlier phases of illness. These results extend our knowledge of the staging model’s assumptions about illness progression and its positive association with certain outcomes overtime. Furthermore, the findings offer support for the use of a multidimensional outcomes framework to facilitate early intervention and inform the allocation of a wider range of specific interventions. Conversely, more research is needed to explain why later stages were not at higher risk of developing alcohol or substance misuse and physical health comorbidities overtime.

One of the main goals of staging and multidimensional assessment is to enhance recovery by personalising interventions to match an individual’s needs, known as staged care [5]. The staged care concept acknowledges that it is socially, economically, and medically justifiable to treat subthreshold disorders to prevent transition to a full threshold illness [34] and to adjust the intensity of interventions based on levels of severity. Previous research shows that failure to assign the appropriate care to an individual may put the young person at risk of illness progression [15] and functional impairment [35]. Therefore, identifying subgroups of individuals who are at greater risk of diverse poor outcomes is important in the allocation of mental health service resources, given their known scarcity in Australia [36]. This study demonstrates that the clinical staging model may help clinicians and health services identify young people at greater risk of poor outcomes.

The emergence of poor functional outcomes (i.e. NEET) was differentiated by the clinical staging model, with those at stages 1b and 2+ having a high risk of progressing from non-NEET to NEET compared to stage 1a. Poor social and occupational functioning is a well-established risk factor for the later development of psychotic disorders [37] and is associated with stage transitions [15], which suggests that poor functioning often precedes mental illness and promotes its progression. Clinical stage also differentiated the relative likelihood of developing circadian disturbances, with later stages representing a greater risk of this outcome. The emergence of sleep-wake cycle disturbances has previously been described as a precursor for mental disorders [38] and predictor of clinical stage transition [15]. The onset of circadian disturbances in later clinical stages may indicate the neurobiological alterations that are shared by severe mental illnesses and sleep disorders [39, 40]. For example, later illness stage is associated with lower right amygdala volume [39] while chronic insomnia disorder is associated with localised amygdala atrophy [40]. Further differentiation of the 1a and 1b groups was substantiated by the comparatively heightened risk of developing suicidal ideations for those in stage 1b. This aligns with prior work that reported a comparable proportion of individuals with suicidal thoughts in stage 1b and 2+ groups [8]. Interestingly, the onset of suicidal ideation did not differ when comparing stage 2+ to the other staging groups. The baseline rates of suicidal ideation between stages 2+ and 1b were also comparable, which suggests that the trajectory of suicidal ideation may not differ for those with attenuated or full-threshold disorders and suggests that the risk of ideation may be established before the development of a full-threshold disorder.

Despite these outcomes contributing to poorer mental and functional outcomes [9, 41–43], targeted interventions can make a difference [5, 44–46]. For example, while behavioural interventions for sleep can be applied across all clinical stages [46], clinicians may need to consider treatments of greater intensity for later clinical stages such as bright light therapy [47], to better manage circadian disturbances for those at later clinical stages. Other specific interventions for suicidal ideation, such as personalised safety plans or dialectical behavioural therapy [45, 48], could be targeted for those at stage 1b given the differential risk compared to those at stage 1a. Taken together, clinical stage may be a key marker to assist clinicians in identifying those with underlying psychopathological risk and inform specialised intervention and secondary prevention. However, the emergence of physical health comorbidities and alcohol or substance misuse did not differ between the clinical staging groups. Given that physical comorbidity is less likely to occur over a shorter 12-month period, this finding is not surprising. The lack of association between alcohol or substance misuse and clinical stage could also be explained by the limited number of individuals that experienced this outcome, or that it has an independent course to illness progression. These findings suggest that allocation of interventions for these domains should not be based simply on clinical stage or syndrome type, but rather facilitated using the multidimensional framework to comprehensively assess needs and provide more personalised or a broader scope of interventions. Thus, the intensity and duration of specific interventions that target functioning, suicidality, and circadian disturbances may need to be stratified by stage, compared to preventative treatments for physical ill-health and alcohol or substance misuse, which may need to be applied to all young people who present for care.

Digital technologies can be used as a scalable solution to facilitate multidimensional assessment at entry into care and for ongoing monitoring, which can promote a change in care plan that is tailored to the individual’s needs [49–51]. Consequently, young people may receive more timely care that prevents the progression of illness and its associated risk of poorer outcomes [49]. Future work should aim to highlight the effect of digitally supported care coordination on longitudinal outcomes in youth mental health, with a particular focus on the multidimensional framework.

Some limitations of this study should be considered alongside our findings. First, this is a naturalistic study that tested our hypotheses in a pre-existing data set. The data were extracted from clinical records as opposed to structured assessments, which may introduce biases in our analyses. An inter-rater analysis found moderate to substantial agreement (kappa range 0.4–0.6) when collecting most data, though estimates varied based on the type of variables being assessed, for example circadian disturbances had a slightly lower agreement with kappa in the fair range (0.2–0.4) [19]. Second, the sample was restricted to those who were followed up at least once over the course of 12 months, which does not represent the entire population of help-seeking youths, particularly as those with complex needs (i.e. in stage 2+) were over-represented in the subpopulations that disengaged from care. Third, this study did not evaluate the effect of any specific interventions that individuals received. Fourth, the current study did not investigate patterns of recovery during the 12-month period. Fifth, we combined stages 2, 3, and 4 into one group (2+) to increase the sample size of the ‘later stage’ group, which may have yielded more pronounced differences compared to the stage 1a and 1b groups. Future studies should focus on those with more advanced clinical stages to establish differential risks among those with established and recurrent disorders. Finally, we did not investigate the role of covariates other than age and follow-up time in the logistic regression models (e.g. medication, at-risk states).

## Conclusions

Overall, this study supports the utility of the clinical staging model to assist in determining differential likelihoods of the emergence of poor multidimensional outcomes over 12 months. Clinical stage was not associated with the longitudinal risk of developing alcohol or substance misuse and physical health comorbidity, which provides insights for further research to clarify whether or not these outcomes emerge independent of stage. By demonstrating the greater risk of developing broader illness impacts for young people with more severe illnesses, these findings support the combined utility of clinical staging and multidimensional assessment in youth mental health care services to guide early intervention and targeted treatment plans.

## Supplementary Information

**Additional file 1.** Contains further detail about the data collection and variables used in this study.

## Data Availability

The datasets used and/or analysed during the current study are available from the corresponding author on reasonable request.

## Abbreviations

ANOVA: Analysis of variance
CI: Confidence interval
DSM: Diagnostic and Statistical Manual of Mental Disorders
NEET: Not in Education, Employment, and Training
OR: Odds ratio
SD: Standard deviation
SOFAS: Social and Occupational Functioning Assessment Scale
