# Supplementary material for: Clinical staging and the differential risks for clinical and functional outcomes in young people presenting for youth mental health care
Source: BMC Med. 2022 Dec 14;20:479. doi: 10.1186/s12916-022-02666-w (PMC9749194; doi:10.1186/s12916-022-02666-w)
Supplement: Supplementary file 1 — Additional file 1. Contains further detail about the data collection and variables used in this study. [file 12916_2022_2666_MOESM1_ESM.docx]

**Additional file 1**

This material has been modified from previous work (1) to give readers additional information about the data collection and variables used in this study.

The clinical notes for all study participants were manually read and assessed by a team of trained clinical researchers. Data was then extracted from these clinical notes by the clinical researchers and entered into the proforma as required. All clinical notes are generated by the study participants’ treating clinician/s as part of their standard care.

*Demographics*

Biological sex, and age. Current engagement in part- or full-time education or employment to determine Not in Education, Employment, or Training (NEET) status. NEET is assigned if there was no full- or part-time education, employment, training, or volunteer work.

*Social and occupational functioning*

The Social and Occupational Functioning Assessment Scale (SOFAS) (2) is a clinician-rated measure that assesses functioning on a 0–100 scale, with lower scores suggesting functional impairment. The instructions emphasise that the assessor should avoid confounding the rating with clinical symptoms (2-4). A SOFAS score of below 70 is considered to be clinically-significant impairment (5).

*Mental disorder diagnoses*

Mental disorder diagnoses at each time point are classified according to Diagnostic and Statistical Manual of Mental Disorders (DSM-5) criteria (6) and specified as either full- or sub-threshold. Diagnoses are also labelled as either primary, secondary, or tertiary based on judgement of which was the dominant presenting problem at that time point.

Mental disorder diagnosis is determined solely by the symptomology and/or diagnosis reported and recorded by the treating clinician/s as presented in the clinical notes of each study participant. Based on the information provided within these clinical notes, researchers determined whether DSM-5 criteria were met for a specific disorder at that time point. If symptomology recorded in the clinical notes indicated only some, but not all criteria being met for a specific disorder, then a sub-threshold classification was recorded. If symptomology indicated full DSM-5 criteria were met for that time point, then a full-threshold classification was recorded.

As per diagnosis, medication is also obtained from a review of the clinical notes as generated by the study participants’ clinician/s. A certain medication is recorded if the clinical notes indicate that the study participant took that particular class of medication within the specified timeframe.

*Clinical stage*

Information about the course of illness is also used to assign a clinical stage at each time point according to a previously established model (7-9) and as described in the introduction and methods. While stages 3 and 4 are also specified elsewhere for recurrent, persistent, and chronic illness courses, stage 2 is the proposed cut-point for more persistent disorders requiring more specific and intensive clinical care and treatment (7-9). With regards to longitudinal assessment, and consistent with other clinical staging models in medicine (e.g. oncology), while an individual may experience clinical remission at any stage, once they reach a certain point on the illness continuum they cannot go back to an earlier stage.

*At-risk mental states*

Clusters of symptoms that have been previously indicated as risk factors for progression to more severe mental disorders (10-15) are recorded in all individuals regardless of diagnosis. This includes psychotic-like experiences (the presence of any psychotic symptoms including: perceptual abnormalities, bizarre ideas, disorganised speech, etc), manic-like experiences (the presence of any manic/hypomanic symptoms including: abnormally elevated mood or irritability; increased motor activity, speech, or sexual interest, etc), and circadian disturbance (the presence of significant disruption in sleep-wake or circadian cycles including the presence of a severe sleep-wake disorder or chronic fatigue). The presence or absence of these clusters of symptoms is determined solely by the symptomology reported and recorded by the treating clinician/s as presented in the clinical notes of each study participant. Similarly, the distinction between psychotic-like and manic-like symptoms is judged within the context of the clinical notes.

The threshold for mania-like experiences and psychotic-like experiences in this study is low. Conversely, the threshold for circadian disturbance in this study is high. More specifically, these experiences are rated based on their presence or absence and the nature (e.g. type, severity, frequency) of these experiences, and so stage 1a and stage 1b mania-like experiences and psychotic-like experiences are not necessarily different, but in some cases may differ in nature. The presence of these symptoms does not necessarily mean the participant currently has / or will go on to develop a serious mental health disorder. It is simply one of many risk factors that may exist. Moreover, the presence or absence of these symptoms do not, in and of themselves, determine the staging of a participant.

*Self-harm and suicidal thoughts and behaviours*

The presence of suicidal ideation, suicide attempts, and self-harm is recorded. A suicide attempt is recorded when a young person has taken steps to take their own life. If an individual harms themselves via cutting, hitting themselves, burning themselves, or scratching with the intention to self-harm only and not to take their life, then this is included as self-harm and not a suicide attempt.

*Physical health comorbidities*

Any major physical illness is recorded.

*Personal mental illness history*

Known childhood-onset disorders (i.e. with clear onset prior to 12 years old) are recorded in addition to current diagnoses. Family history of a mental health disorder is ascertained via the treating clinician’s assessment with the client. Please note, family history is only recorded when the client has reported a mental health diagnosis of a first degree relative. Moreover, family history is only recorded if the client reported that the first-degree family member has a current of past diagnosed mental health disorder. Symptoms only, but no diagnosis, is not enough to meet criteria for this category.

*Treatment utilisation*

Exposure to classes of medication (antidepressant, antipsychotic, mood stabiliser, or stimulant medication), and hospitalisation overnight or longer due to a mental health problem are recorded.

1. Iorfino F, Scott EM, Carpenter JS, Cross SP, Hermens DF, Killedar M, et al. Clinical stage transitions in persons aged 12 to 25 years presenting to early intervention mental health services with anxiety, mood, and psychotic Disorders. JAMA Psychiatry. 2019;76(11):1167-75.

2. Goldman HH, Skodol AE, Lave TR. Revising axis V for DSM-IV: a review of measures of social functioning. Am J Psychiatry. 1992;149(9):1148-56.

3. Hilsenroth MJ, Ackerman SJ, Blagys MD, Baumann BD, Baity MR, Smith SR, et al. Reliability and Validity of DSM-IV Axis V. American Journal of Psychiatry. 2000;157:1858-63.

4. Hay P, Katsikitis M, Begg J, Da Costa J, Blumenfeld N. A Two-Year Follow-Up Study and Prospective Evaluation of the DSM-IV Axis V. Psychiatric Services. 2003;51:1028-30.

5. Rickwood DJ, Mazzer KR, Telford NR, Parker AG, Tanti CJ, McGorry PD. Changes in psychological distress and psychosocial functioning in young people visiting headspace centres for mental health problems. Med J Aust. 2015;202(10):537-42.

6. American Psychiatric Association. Diagnostic and statistical manual of mental disorders. 5th ed. Arlington, VA2013.

7. Hickie IB, Scott EM, Hermens DF, Naismith SL, Guastella AJ, Kaur M, et al. Applying clinical staging to young people who present for mental health care. Early Interv Psychiatry. 2013;7(1):31-43.

8. McGorry PD, Hickie IB, Yung AR, Pantelis C, Jackson HJ. Clinical staging of psychiatric disorders: A heuristic framework for choosing earlier, safer and more effective interventions. Aust N Z J Psychiatry. 2006;40:616-22.

9. McGorry PD, Purcell R, Hickie IB, Yung AR, Pantelis C, Jackson HJ. Clinical staging: A heuristic model for psychiatry and youth mental health. Medical Journal of Australia. 2007;187:S40-S2.

10. Kelleher I, Keeley H, Corcoran P, Lynch F, Fitzpatrick C, Devlin N, et al. Clinicopathological significance of psychotic experiences in non-psychotic young people: Evidence from four population-based studies. British Journal of Psychiatry. 2012;201(1):26-32.

11. Kelleher I, Cannon M. Psychotic-like experiences in the general population: characterizing a high-risk group for psychosis. Psychol Med. 2011;41(1):1-6.

12. Hauser M, Correll CU. The Significance of At-Risk or Prodromal Symptoms for Bipolar I Disorder in Children and Adolescents. Canadian Journal of Psychiatry. 2013;58(1):22-31.

13. Faedda GL, Marangoni C, Serra G, Salvatore P, Sani G, Vazquez GH, et al. Precursors of bipolar disorders: A systematic literature review of prospective studies. The Journal of clinical psychiatry. 2015;76(5):614-24.

14. Alvaro PK, Roberts RM, Harris JK. A systematic review assessing bidirectionality between sleep disturbances, anxiety, and depression. Sleep. 2013;36(7):1059-68.

15. Addington AM, Gallo JJ, Ford DE, Eaton WW. Epidemiology of unexplained fatigue and major depression in the community: The Baltimore ECA Follow-up, 1981–1994. Psychological Medicine. 2001;31(6):1037-44.
